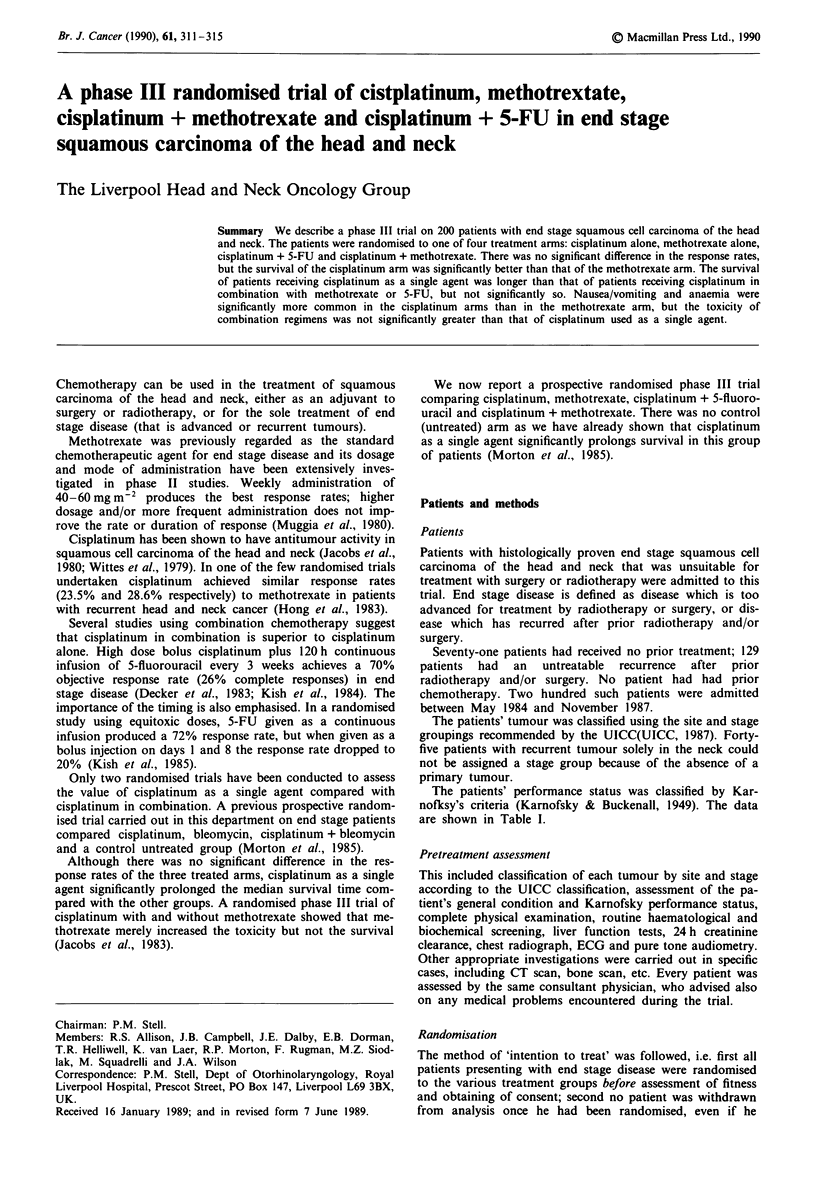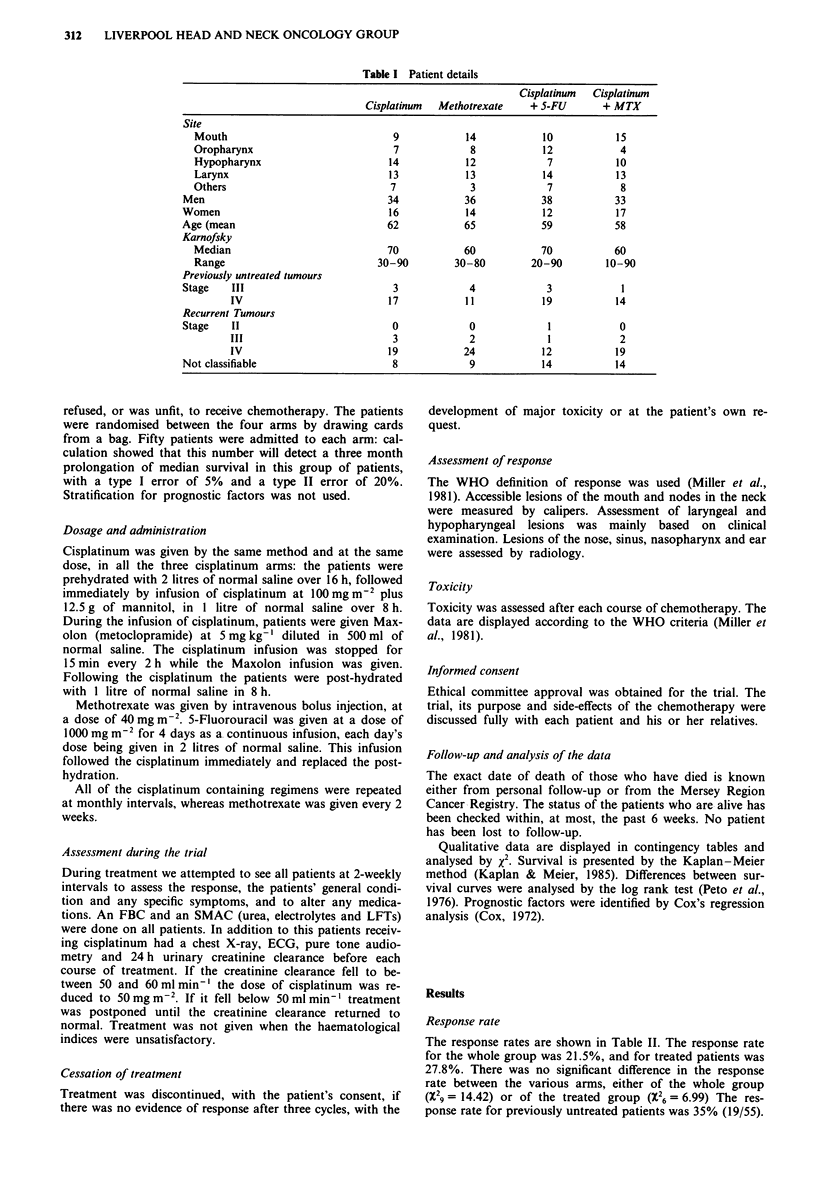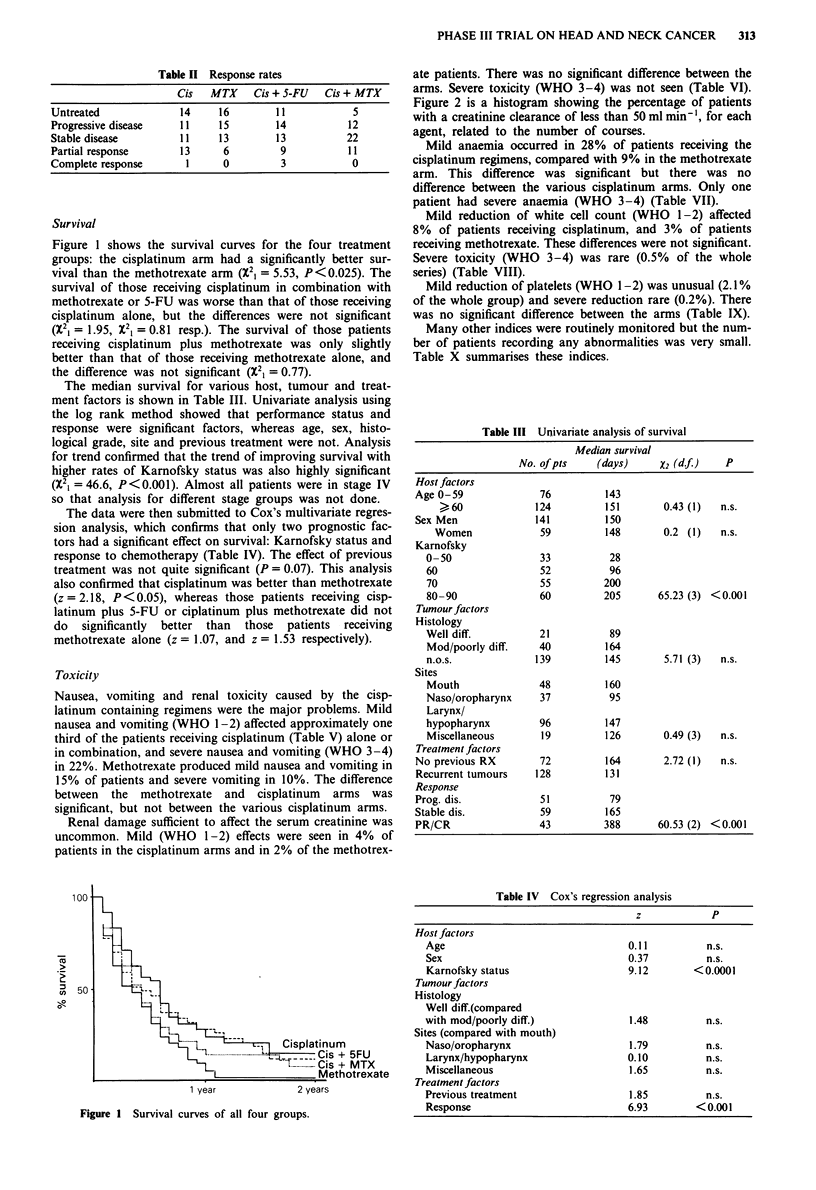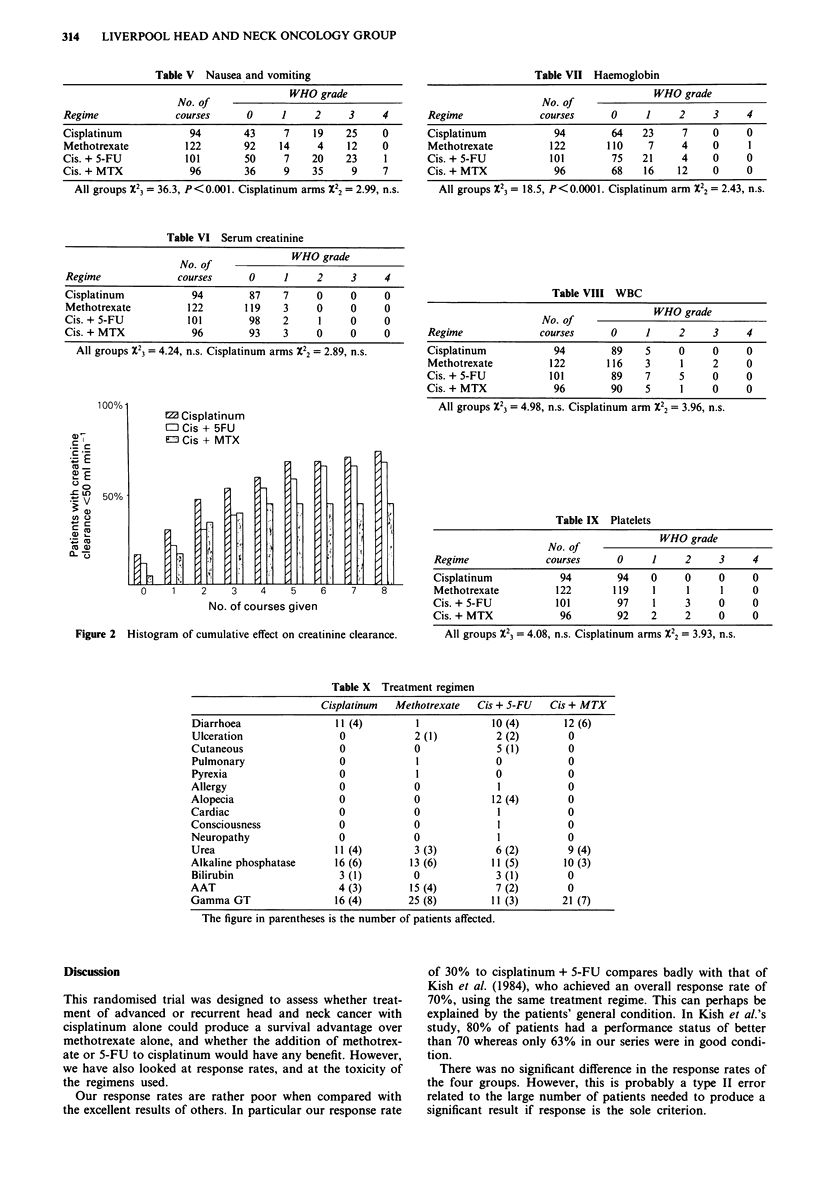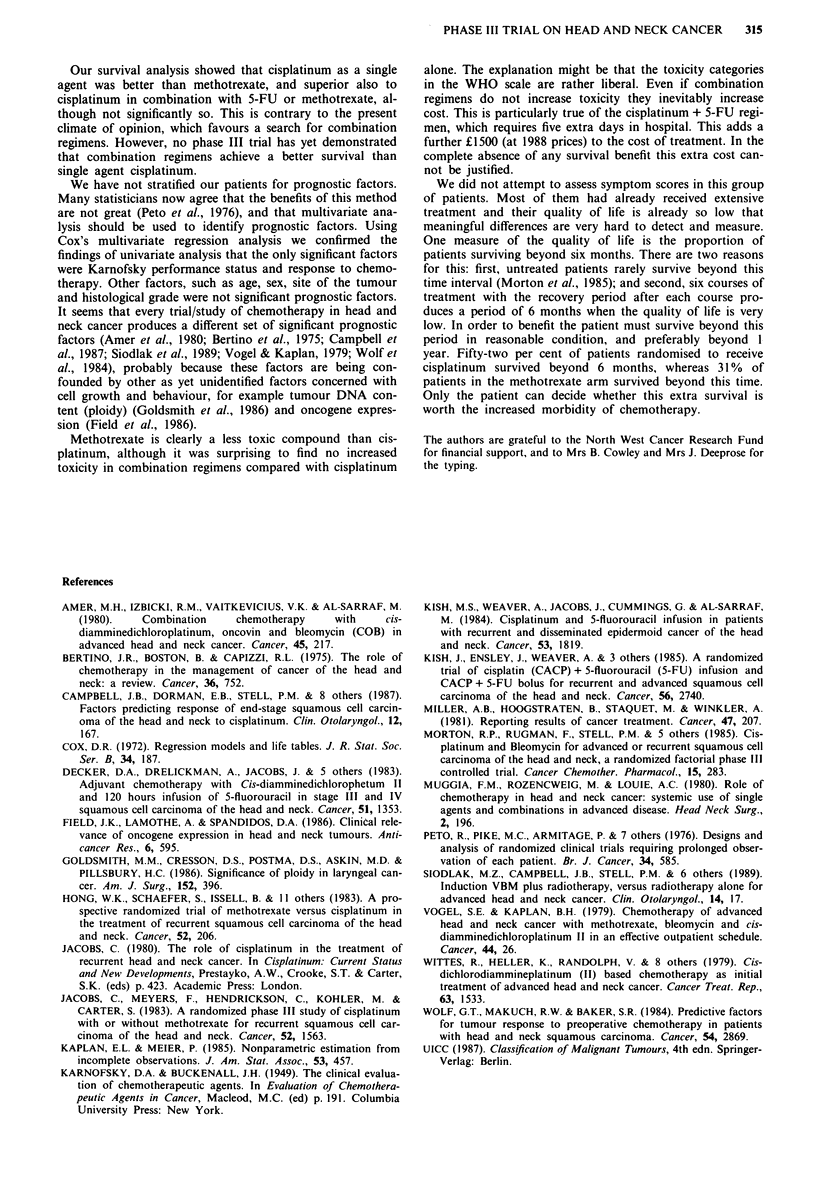# A phase III randomised trial of cisplatinum, methotrextate, cisplatinum + methotrexate and cisplatinum + 5-FU in end stage squamous carcinoma of the head and neck. Liverpool Head and Neck Oncology Group.

**DOI:** 10.1038/bjc.1990.59

**Published:** 1990-02

**Authors:** 

## Abstract

We describe a phase III trial on 200 patients with end stage squamous cell carcinoma of the head and neck. The patients were randomised to one of four treatment arms: cisplatinum alone, methotrexate alone, cisplatinum + 5-FU and cisplatinum + methotrexate. There was no significant difference in the response rates, but the survival of the cisplatinum arm was significantly better than that of the methotrexate arm. The survival of patients receiving cisplatinum as a single agent was longer than that of patients receiving cisplatinum in combination with methotrexate or 5-FU, but not significantly so. Nausea/vomiting and anaemia were significantly more common in the cisplatinum arms than in the methotrexate arm, but the toxicity of combination regimens was not significantly greater than that of cisplatinum used as a single agent.


Br. J. Cancer (1990), 61, 311-315 © Macmillan Press Ltd., 1990

### A phase III randomised trial of cistplatinum, methotrextate, cisplatinum + methotrexate and cisplatinum + 5-FU in end stage squamous carcinoma of the head and neck

The Liverpool Head and Neck Oncology Group

**Summary** We describe a phase III trial on 200 patients with end stage squamous cell carcinoma of the head and neck. The patients were randomised to one of four treatment arms: cisplatinum alone, methotrexate alone, cisplatinum + 5-FU and cisplatinum + methotrexate. There was no significant difference in the response rates, but the survival of the cisplatinum arm was significantly better than that of the methotrexate arm. The survival of patients receiving cisplatinum as a single agent was longer than that of patients receiving cisplatinum in combination with methotrexate or 5-FU, but not significantly so. Nausea/vomiting and anaemia were significantly more common in the cisplatinum arms than in the methotrexate arm, but the toxicity of combination regimens was not significantly greater than that of cisplatinum used as a single agent.

Chemotherapy can be used in the treatment of squamous carcinoma of the head and neck, either as an adjuvant to surgery or radiotherapy, or for the sole treatment of end stage disease (that is advanced or recurrent tumours).

Methotrexate was previously regarded as the standard chemotherapeutic agent for end stage disease and its dosage and mode of administration have been extensively investigated in phase II studies. Weekly administration of 40-60 $mg\,m^{-2}$ produces the best response rates; higher dosage and/or more frequent administration does not improve the rate or duration of response (Muggia *et al.*, 1980).

Cisplatinum has been shown to have antitumour activity in squamous cell carcinoma of the head and neck (Jacobs *et al.*, 1980; Wittes *et al.*, 1979). In one of the few randomised trials undertaken cisplatinum achieved similar response rates (23.5% and 28.6% respectively) to methotrexate in patients with recurrent head and neck cancer (Hong *et al.*, 1983).

Several studies using combination chemotherapy suggest that cisplatinum in combination is superior to cisplatinum alone. High dose bolus cisplatinum plus 120 h continuous infusion of 5-fluorouracil every 3 weeks achieves a 70% objective response rate (26% complete responses) in end stage disease (Decker *et al.*, 1983; Kish *et al.*, 1984). The importance of the timing is also emphasised. In a randomised study using equitoxic doses, 5-FU given as a continuous infusion produced a 72% response rate, but when given as a bolus injection on days 1 and 8 the response rate dropped to 20% (Kish *et al.*, 1985).

Only two randomised trials have been conducted to assess the value of cisplatinum as a single agent compared with cisplatinum in combination. A previous prospective randomised trial carried out in this department on end stage patients compared cisplatinum, bleomycin, cisplatinum + bleomycin and a control untreated group (Morton *et al.*, 1985).

Although there was no significant difference in the response rates of the three treated arms, cisplatinum as a single agent significantly prolonged the median survival time compared with the other groups. A randomised phase III trial of cisplatinum with and without methotrexate showed that methotrexate merely increased the toxicity but not the survival (Jacobs *et al.*, 1983).

Chairman: P.M. Stell.
Members: R.S. Allison, J.B. Campbell, J.E. Dalby, E.B. Dorman, T.R. Helliwell, K. van Laer, R.P. Morton, F. Rugman, M.Z. Siodlak, M. Squadrelli and J.A. Wilson
Correspondence: P.M. Stell, Dept of Otorhinolaryngology, Royal Liverpool Hospital, Prescot Street, PO Box 147, Liverpool L69 3BX, UK.
Received 16 January 1989; and in revised form 7 June 1989.

We now report a prospective randomised phase III trial comparing cisplatinum, methotrexate, cisplatinum + 5-fluorouracil and cisplatinum + methotrexate. There was no control (untreated) arm as we have already shown that cisplatinum as a single agent significantly prolongs survival in this group of patients (Morton *et al.*, 1985).

### Patients and methods

### *Patients*

Patients with histologically proven end stage squamous cell carcinoma of the head and neck that was unsuitable for treatment with surgery or radiotherapy were admitted to this trial. End stage disease is defined as disease which is too advanced for treatment by radiotherapy or surgery, or disease which has recurred after prior radiotherapy and/or surgery.

Seventy-one patients had received no prior treatment; 129 patients had an untreatable recurrence after prior radiotherapy and/or surgery. No patient had had prior chemotherapy. Two hundred such patients were admitted between May 1984 and November 1987.

The patients' tumour was classified using the site and stage groupings recommended by the UICC(UICC, 1987). Forty-five patients with recurrent tumour solely in the neck could not be assigned a stage group because of the absence of a primary tumour.

The patients' performance status was classified by Karnofksy's criteria (Karnofsky & Buckenall, 1949). The data are shown in Table I.

### *Pretreatment assessment*

This included classification of each tumour by site and stage according to the UICC classification, assessment of the patient's general condition and Karnofsky performance status, complete physical examination, routine haematological and biochemical screening, liver function tests, 24 h creatinine clearance, chest radiograph, ECG and pure tone audiometry. Other appropriate investigations were carried out in specific cases, including CT scan, bone scan, etc. Every patient was assessed by the same consultant physician, who advised also on any medical problems encountered during the trial.

### *Randomisation*

The method of 'intention to treat' was followed, i.e. first all patients presenting with end stage disease were randomised to the various treatment groups *before* assessment of fitness and obtaining of consent; second no patient was withdrawn from analysis once he had been randomised, even if he

**Table I** Patient details

| | *Cisplatinum* | *Methotrexate* | *Cisplatinum + 5-FU* | *Cisplatinum + MTX* |
|---|---|---|---|---|
| *Site* | | | | |
| Mouth | 9 | 14 | 10 | 15 |
| Oropharynx | 7 | 8 | 12 | 4 |
| Hypopharynx | 14 | 12 | 7 | 10 |
| Larynx | 13 | 13 | 14 | 13 |
| Others | 7 | 3 | 7 | 8 |
| Men | 34 | 36 | 38 | 33 |
| Women | 16 | 14 | 12 | 17 |
| Age (mean | 62 | 65 | 59 | 58 |
| *Karnofsky* | | | | |
| Median | 70 | 60 | 70 | 60 |
| Range | 30–90 | 30–80 | 20–90 | 10–90 |
| *Previously untreated tumours* | | | | |
| Stage III | 3 | 4 | 3 | 1 |
| IV | 17 | 11 | 19 | 14 |
| *Recurrent Tumours* | | | | |
| Stage II | 0 | 0 | 1 | 0 |
| III | 3 | 2 | 1 | 2 |
| IV | 19 | 24 | 12 | 19 |
| Not classifiable | 8 | 9 | 14 | 14 |

refused, or was unfit, to receive chemotherapy. The patients were randomised between the four arms by drawing cards from a bag. Fifty patients were admitted to each arm: calculation showed that this number will detect a three month prolongation of median survival in this group of patients, with a type I error of 5% and a type II error of 20%. Stratification for prognostic factors was not used.

### *Dosage and administration*

Cisplatinum was given by the same method and at the same dose, in all the three cisplatinum arms: the patients were prehydrated with 2 litres of normal saline over 16 h, followed immediately by infusion of cisplatinum at $100\,mg\,m^{-2}$ plus 12.5 g of mannitol, in 1 litre of normal saline over 8 h. During the infusion of cisplatinum, patients were given Maxolon (metoclopramide) at $5\,mg\,kg^{-1}$ diluted in 500 ml of normal saline. The cisplatinum infusion was stopped for 15 min every 2 h while the Maxolon infusion was given. Following the cisplatinum the patients were post-hydrated with 1 litre of normal saline in 8 h.

Methotrexate was given by intravenous bolus injection, at a dose of $40\,mg\,m^{-2}$. 5-Fluorouracil was given at a dose of $1000\,mg\,m^{-2}$ for 4 days as a continuous infusion, each day's dose being given in 2 litres of normal saline. This infusion followed the cisplatinum immediately and replaced the post-hydration.

All of the cisplatinum containing regimens were repeated at monthly intervals, whereas methotrexate was given every 2 weeks.

### *Assessment during the trial*

During treatment we attempted to see all patients at 2-weekly intervals to assess the response, the patients' general condition and any specific symptoms, and to alter any medications. An FBC and an SMAC (urea, electrolytes and LFTs) were done on all patients. In addition to this patients receiving cisplatinum had a chest X-ray, ECG, pure tone audiometry and 24 h urinary creatinine clearance before each course of treatment. If the creatinine clearance fell to between 50 and $60\,ml\,min^{-1}$ the dose of cisplatinum was reduced to $50\,mg\,m^{-2}$. If it fell below $50\,ml\,min^{-1}$ treatment was postponed until the creatinine clearance returned to normal. Treatment was not given when the haematological indices were unsatisfactory.

### *Cessation of treatment*

Treatment was discontinued, with the patient's consent, if there was no evidence of response after three cycles, with the development of major toxicity or at the patient's own request.

### *Assessment of response*

The WHO definition of response was used (Miller *et al.*, 1981). Accessible lesions of the mouth and nodes in the neck were measured by calipers. Assessment of laryngeal and hypopharyngeal lesions was mainly based on clinical examination. Lesions of the nose, sinus, nasopharynx and ear were assessed by radiology.

### *Toxicity*

Toxicity was assessed after each course of chemotherapy. The data are displayed according to the WHO criteria (Miller *et al.*, 1981).

### *Informed consent*

Ethical committee approval was obtained for the trial. The trial, its purpose and side-effects of the chemotherapy were discussed fully with each patient and his or her relatives.

### *Follow-up and analysis of the data*

The exact date of death of those who have died is known either from personal follow-up or from the Mersey Region Cancer Registry. The status of the patients who are alive has been checked within, at most, the past 6 weeks. No patient has been lost to follow-up.

Qualitative data are displayed in contingency tables and analysed by $\chi^2$. Survival is presented by the Kaplan–Meier method (Kaplan & Meier, 1985). Differences between survival curves were analysed by the log rank test (Peto *et al.*, 1976). Prognostic factors were identified by Cox's regression analysis (Cox, 1972).

### Results

### *Response rate*

The response rates are shown in Table II. The response rate for the whole group was 21.5%, and for treated patients was 27.8%. There was no significant difference in the response rate between the various arms, either of the whole group ($\chi^2_9 = 14.42$) or of the treated group ($\chi^2_6 = 6.99$) The response rate for previously untreated patients was 35% (19/55).

**Table II** Response rates

| | *Cis* | *MTX* | *Cis + 5-FU* | *Cis + MTX* |
|---|---|---|---|---|
| Untreated | 14 | 16 | 11 | 5 |
| Progressive disease | 11 | 15 | 14 | 12 |
| Stable disease | 11 | 13 | 13 | 22 |
| Partial response | 13 | 6 | 9 | 11 |
| Complete response | 1 | 0 | 3 | 0 |

### *Survival*

Figure 1 shows the survival curves for the four treatment groups: the cisplatinum arm had a significantly better survival than the methotrexate arm ($\chi^2_1 = 5.53$, $P<0.025$). The survival of those receiving cisplatinum in combination with methotrexate or 5-FU was worse than that of those receiving cisplatinum alone, but the differences were not significant ($\chi^2_1 = 1.95$, $\chi^2_1 = 0.81$ resp.). The survival of those patients receiving cisplatinum plus methotrexate was only slightly better than that of those receiving methotrexate alone, and the difference was not significant ($\chi^2_1 = 0.77$).

The median survival for various host, tumour and treatment factors is shown in Table III. Univariate analysis using the log rank method showed that performance status and response were significant factors, whereas age, sex, histological grade, site and previous treatment were not. Analysis for trend confirmed that the trend of improving survival with higher rates of Karnofsky status was also highly significant ($\chi^2_1 = 46.6$, $P<0.001$). Almost all patients were in stage IV so that analysis for different stage groups was not done.

The data were then submitted to Cox's multivariate regression analysis, which confirms that only two prognostic factors had a significant effect on survival: Karnofsky status and response to chemotherapy (Table IV). The effect of previous treatment was not quite significant ($P = 0.07$). This analysis also confirmed that cisplatinum was better than methotrexate ($z = 2.18$, $P<0.05$), whereas those patients receiving cisplatinum plus 5-FU or ciplatinum plus methotrexate did not do significantly better than those patients receiving methotrexate alone ($z = 1.07$, and $z = 1.53$ respectively).

### *Toxicity*

Nausea, vomiting and renal toxicity caused by the cisplatinum containing regimens were the major problems. Mild nausea and vomiting (WHO 1–2) affected approximately one third of the patients receiving cisplatinum (Table V) alone or in combination, and severe nausea and vomiting (WHO 3–4) in 22%. Methotrexate produced mild nausea and vomiting in 15% of patients and severe vomiting in 10%. The difference between the methotrexate and cisplatinum arms was significant, but not between the various cisplatinum arms.

Renal damage sufficient to affect the serum creatinine was uncommon. Mild (WHO 1–2) effects were seen in 4% of patients in the cisplatinum arms and in 2% of the methotrexate patients. There was no significant difference between the arms. Severe toxicity (WHO 3–4) was not seen (Table VI). Figure 2 is a histogram showing the percentage of patients with a creatinine clearance of less than 50 ml $min^{-1}$, for each agent, related to the number of courses.

Mild anaemia occurred in 28% of patients receiving the cisplatinum regimens, compared with 9% in the methotrexate arm. This difference was significant but there was no difference between the various cisplatinum arms. Only one patient had severe anaemia (WHO 3–4) (Table VII).

Mild reduction of white cell count (WHO 1–2) affected 8% of patients receiving cisplatinum, and 3% of patients receiving methotrexate. These differences were not significant. Severe toxicity (WHO 3–4) was rare (0.5% of the whole series) (Table VIII).

Mild reduction of platelets (WHO 1–2) was unusual (2.1% of the whole group) and severe reduction rare (0.2%). There was no significant difference between the arms (Table IX).

Many other indices were routinely monitored but the number of patients recording any abnormalities was very small. Table X summarises these indices.

**Table III** Univariate analysis of survival

| | *No. of pts* | *Median survival (days)* | $\chi_2$ *(d.f.)* | *P* |
|---|---|---|---|---|
| *Host factors* | | | | |
| Age 0–59 | 76 | 143 | | |
| ⩾60 | 124 | 151 | 0.43 (1) | n.s. |
| Sex Men | 141 | 150 | | |
| Women | 59 | 148 | 0.2 (1) | n.s. |
| Karnofsky | | | | |
| 0–50 | 33 | 28 | | |
| 60 | 52 | 96 | | |
| 70 | 55 | 200 | | |
| 80–90 | 60 | 205 | 65.23 (3) | <0.001 |
| *Tumour factors* | | | | |
| Histology | | | | |
| Well diff. | 21 | 89 | | |
| Mod/poorly diff. | 40 | 164 | | |
| n.o.s. | 139 | 145 | 5.71 (3) | n.s. |
| Sites | | | | |
| Mouth | 48 | 160 | | |
| Naso/oropharynx | 37 | 95 | | |
| Larynx/ hypopharynx | 96 | 147 | | |
| Miscellaneous | 19 | 126 | 0.49 (3) | n.s. |
| *Treatment factors* | | | | |
| No previous RX | 72 | 164 | 2.72 (1) | n.s. |
| Recurrent tumours | 128 | 131 | | |
| *Response* | | | | |
| Prog. dis. | 51 | 79 | | |
| Stable dis. | 59 | 165 | | |
| PR/CR | 43 | 388 | 60.53 (2) | <0.001 |

**Figure 1** Survival curves of all four groups.

**Table IV** Cox's regression analysis

| | *z* | *P* |
|---|---|---|
| *Host factors* | | |
| Age | 0.11 | n.s. |
| Sex | 0.37 | n.s. |
| Karnofsky status | 9.12 | <0.0001 |
| *Tumour factors* | | |
| Histology | | |
| Well diff.(compared with mod/poorly diff.) | 1.48 | n.s. |
| Sites (compared with mouth) | | |
| Naso/oropharynx | 1.79 | n.s. |
| Larynx/hypopharynx | 0.10 | n.s. |
| Miscellaneous | 1.65 | n.s. |
| *Treatment factors* | | |
| Previous treatment | 1.85 | n.s. |
| Response | 6.93 | <0.001 |

**Table V** Nausea and vomiting

| Regime | No. of courses | WHO grade 0 | 1 | 2 | 3 | 4 |
|---|---|---|---|---|---|---|
| Cisplatinum | 94 | 43 | 7 | 19 | 25 | 0 |
| Methotrexate | 122 | 92 | 14 | 4 | 12 | 0 |
| Cis. + 5-FU | 101 | 50 | 7 | 20 | 23 | 1 |
| Cis. + MTX | 96 | 36 | 9 | 35 | 9 | 7 |

All groups $\chi^2_3 = 36.3$, $P < 0.001$. Cisplatinum arms $\chi^2_2 = 2.99$, n.s.

**Table VI** Serum creatinine

| Regime | No. of courses | WHO grade 0 | 1 | 2 | 3 | 4 |
|---|---|---|---|---|---|---|
| Cisplatinum | 94 | 87 | 7 | 0 | 0 | 0 |
| Methotrexate | 122 | 119 | 3 | 0 | 0 | 0 |
| Cis. + 5-FU | 101 | 98 | 2 | 1 | 0 | 0 |
| Cis. + MTX | 96 | 93 | 3 | 0 | 0 | 0 |

All groups $\chi^2_3 = 4.24$, n.s. Cisplatinum arms $\chi^2_2 = 2.89$, n.s.

**Figure 2** Histogram of cumulative effect on creatinine clearance.

**Table VII** Haemoglobin

| Regime | No. of courses | WHO grade 0 | 1 | 2 | 3 | 4 |
|---|---|---|---|---|---|---|
| Cisplatinum | 94 | 64 | 23 | 7 | 0 | 0 |
| Methotrexate | 122 | 110 | 7 | 4 | 0 | 1 |
| Cis. + 5-FU | 101 | 75 | 21 | 4 | 0 | 0 |
| Cis. + MTX | 96 | 68 | 16 | 12 | 0 | 0 |

All groups $\chi^2_3 = 18.5$, $P < 0.0001$. Cisplatinum arm $\chi^2_2 = 2.43$, n.s.

**Table VIII** WBC

| Regime | No. of courses | WHO grade 0 | 1 | 2 | 3 | 4 |
|---|---|---|---|---|---|---|
| Cisplatinum | 94 | 89 | 5 | 0 | 0 | 0 |
| Methotrexate | 122 | 116 | 3 | 1 | 2 | 0 |
| Cis. + 5-FU | 101 | 89 | 7 | 5 | 0 | 0 |
| Cis. + MTX | 96 | 90 | 5 | 1 | 0 | 0 |

All groups $\chi^2_3 = 4.98$, n.s. Cisplatinum arm $\chi^2_2 = 3.96$, n.s.

**Table IX** Platelets

| Regime | No. of courses | WHO grade 0 | 1 | 2 | 3 | 4 |
|---|---|---|---|---|---|---|
| Cisplatinum | 94 | 94 | 0 | 0 | 0 | 0 |
| Methotrexate | 122 | 119 | 1 | 1 | 1 | 0 |
| Cis. + 5-FU | 101 | 97 | 1 | 3 | 0 | 0 |
| Cis. + MTX | 96 | 92 | 2 | 2 | 0 | 0 |

All groups $\chi^2_3 = 4.08$, n.s. Cisplatinum arms $\chi^2_2 = 3.93$, n.s.

**Table X** Treatment regimen

| | Cisplatinum | Methotrexate | Cis + 5-FU | Cis + MTX |
|---|---|---|---|---|
| Diarrhoea | 11 (4) | 1 | 10 (4) | 12 (6) |
| Ulceration | 0 | 2 (1) | 2 (2) | 0 |
| Cutaneous | 0 | 0 | 5 (1) | 0 |
| Pulmonary | 0 | 1 | 0 | 0 |
| Pyrexia | 0 | 1 | 0 | 0 |
| Allergy | 0 | 0 | 1 | 0 |
| Alopecia | 0 | 0 | 12 (4) | 0 |
| Cardiac | 0 | 0 | 1 | 0 |
| Consciousness | 0 | 0 | 1 | 0 |
| Neuropathy | 0 | 0 | 1 | 0 |
| Urea | 11 (4) | 3 (3) | 6 (2) | 9 (4) |
| Alkaline phosphatase | 16 (6) | 13 (6) | 11 (5) | 10 (3) |
| Bilirubin | 3 (1) | 0 | 3 (1) | 0 |
| AAT | 4 (3) | 15 (4) | 7 (2) | 0 |
| Gamma GT | 16 (4) | 25 (8) | 11 (3) | 21 (7) |

The figure in parentheses is the number of patients affected.

### Discussion

This randomised trial was designed to assess whether treatment of advanced or recurrent head and neck cancer with cisplatinum alone could produce a survival advantage over methotrexate alone, and whether the addition of methotrexate or 5-FU to cisplatinum would have any benefit. However, we have also looked at response rates, and at the toxicity of the regimens used.

Our response rates are rather poor when compared with the excellent results of others. In particular our response rate of 30% to cisplatinum + 5-FU compares badly with that of Kish *et al.* (1984), who achieved an overall response rate of 70%, using the same treatment regime. This can perhaps be explained by the patients' general condition. In Kish *et al.*'s study, 80% of patients had a performance status of better than 70 whereas only 63% in our series were in good condition.

There was no significant difference in the response rates of the four groups. However, this is probably a type II error related to the large number of patients needed to produce a significant result if response is the sole criterion.

Our survival analysis showed that cisplatinum as a single agent was better than methotrexate, and superior also to cisplatinum in combination with 5-FU or methotrexate, although not significantly so. This is contrary to the present climate of opinion, which favours a search for combination regimens. However, no phase III trial has yet demonstrated that combination regimens achieve a better survival than single agent cisplatinum.

We have not stratified our patients for prognostic factors. Many statisticians now agree that the benefits of this method are not great (Peto *et al.*, 1976), and that multivariate analysis should be used to identify prognostic factors. Using Cox's multivariate regression analysis we confirmed the findings of univariate analysis that the only significant factors were Karnofsky performance status and response to chemotherapy. Other factors, such as age, sex, site of the tumour and histological grade were not significant prognostic factors. It seems that every trial/study of chemotherapy in head and neck cancer produces a different set of significant prognostic factors (Amer *et al.*, 1980; Bertino *et al.*, 1975; Campbell *et al.*, 1987; Siodlak *et al.*, 1989; Vogel & Kaplan, 1979; Wolf *et al.*, 1984), probably because these factors are being confounded by other as yet unidentified factors concerned with cell growth and behaviour, for example tumour DNA content (ploidy) (Goldsmith *et al.*, 1986) and oncogene expression (Field *et al.*, 1986).

Methotrexate is clearly a less toxic compound than cisplatinum, although it was surprising to find no increased toxicity in combination regimens compared with cisplatinum alone. The explanation might be that the toxicity categories in the WHO scale are rather liberal. Even if combination regimens do not increase toxicity they inevitably increase cost. This is particularly true of the cisplatinum + 5-FU regimen, which requires five extra days in hospital. This adds a further £1500 (at 1988 prices) to the cost of treatment. In the complete absence of any survival benefit this extra cost cannot be justified.

We did not attempt to assess symptom scores in this group of patients. Most of them had already received extensive treatment and their quality of life is already so low that meaningful differences are very hard to detect and measure. One measure of the quality of life is the proportion of patients surviving beyond six months. There are two reasons for this: first, untreated patients rarely survive beyond this time interval (Morton *et al.*, 1985); and second, six courses of treatment with the recovery period after each course produces a period of 6 months when the quality of life is very low. In order to benefit the patient must survive beyond this period in reasonable condition, and preferably beyond 1 year. Fifty-two per cent of patients randomised to receive cisplatinum survived beyond 6 months, whereas 31% of patients in the methotrexate arm survived beyond this time. Only the patient can decide whether this extra survival is worth the increased morbidity of chemotherapy.

The authors are grateful to the North West Cancer Research Fund for financial support, and to Mrs B. Cowley and Mrs J. Deeprose for the typing.

### References

AMER, M.H., IZBICKI, R.M., VAITKEVICIUS, V.K. & AL-SARRAF, M. (1980). Combination chemotherapy with *cis*-diamminedichloroplatinum, oncovin and bleomycin (COB) in advanced head and neck cancer. *Cancer*, **45**, 217.

BERTINO, J.R., BOSTON, B. & CAPIZZI, R.L. (1975). The role of chemotherapy in the management of cancer of the head and neck: a review. *Cancer*, **36**, 752.

CAMPBELL, J.B., DORMAN, E.B., STELL, P.M. & 8 others (1987). Factors predicting response of end-stage squamous cell carcinoma of the head and neck to cisplatinum. *Clin. Otolaryngol.*, **12**, 167.

COX, D.R. (1972). Regression models and life tables. *J. R. Stat. Soc. Ser. B*, **34**, 187.

DECKER, D.A., DRELICKMAN, A., JACOBS, J. & 5 others (1983). Adjuvant chemotherapy with *Cis*-diamminedichlorophetum II and 120 hours infusion of 5-fluorouracil in stage III and IV squamous cell carcinoma of the head and neck. *Cancer*, **51**, 1353.

FIELD, J.K., LAMOTHE, A. & SPANDIDOS, D.A. (1986). Clinical relevance of oncogene expression in head and neck tumours. *Anticancer Res.*, **6**, 595.

GOLDSMITH, M.M., CRESSON, D.S., POSTMA, D.S., ASKIN, M.D. & PILLSBURY, H.C. (1986). Significance of ploidy in laryngeal cancer. *Am. J. Surg.*, **152**, 396.

HONG, W.K., SCHAEFER, S., ISSELL, B. & 11 others (1983). A prospective randomized trial of methotrexate versus cisplatinum in the treatment of recurrent squamous cell carcinoma of the head and neck. *Cancer*, **52**, 206.

JACOBS, C. (1980). The role of cisplatinum in the treatment of recurrent head and neck cancer. In *Cisplatinum: Current Status and New Developments*, Prestayko, A.W., Crooke, S.T. & Carter, S.K. (eds) p. 423. Academic Press: London.

JACOBS, C., MEYERS, F., HENDRICKSON, C., KOHLER, M. & CARTER, S. (1983). A randomized phase III study of cisplatinum with or without methotrexate for recurrent squamous cell carcinoma of the head and neck. *Cancer*, **52**, 1563.

KAPLAN, E.L. & MEIER, P. (1985). Nonparametric estimation from incomplete observations. *J. Am. Stat. Assoc.*, **53**, 457.

KARNOFSKY, D.A. & BUCKENALL, J.H. (1949). The clinical evaluation of chemotherapeutic agents. In *Evaluation of Chemotherapeutic Agents in Cancer*, Macleod, M.C. (ed) p. 191. Columbia University Press: New York.

KISH, M.S., WEAVER, A., JACOBS, J., CUMMINGS, G. & AL-SARRAF, M. (1984). Cisplatinum and 5-fluorouracil infusion in patients with recurrent and disseminated epidermoid cancer of the head and neck. *Cancer*, **53**, 1819.

KISH, J., ENSLEY, J., WEAVER, A. & 3 others (1985). A randomized trial of cisplatin (CACP) + 5-fluorouracil (5-FU) infusion and CACP + 5-FU bolus for recurrent and advanced squamous cell carcinoma of the head and neck. *Cancer*, **56**, 2740.

MILLER, A.B., HOOGSTRATEN, B., STAQUET, M. & WINKLER, A. (1981). Reporting results of cancer treatment. *Cancer*, **47**, 207.

MORTON, R.P., RUGMAN, F., STELL, P.M. & 5 others (1985). Cisplatinum and Bleomycin for advanced or recurrent squamous cell carcinoma of the head and neck, a randomized factorial phase III controlled trial. *Cancer Chemother. Pharmacol.*, **15**, 283.

MUGGIA, F.M., ROZENCWEIG, M. & LOUIE, A.C. (1980). Role of chemotherapy in head and neck cancer: systemic use of single agents and combinations in advanced disease. *Head Neck Surg.*, **2**, 196.

PETO, R., PIKE, M.C., ARMITAGE, P. & 7 others (1976). Designs and analysis of randomized clinical trials requiring prolonged observation of each patient. *Br. J. Cancer*, **34**, 585.

SIODLAK, M.Z., CAMPBELL, J.B., STELL, P.M. & 6 others (1989). Induction VBM plus radiotherapy, versus radiotherapy alone for advanced head and neck cancer. *Clin. Otolaryngol.*, **14**, 17.

VOGEL, S.E. & KAPLAN, B.H. (1979). Chemotherapy of advanced head and neck cancer with methotrexate, bleomycin and *cis*-diamminedichloroplatinum II in an effective outpatient schedule. *Cancer*, **44**, 26.

WITTES, R., HELLER, K., RANDOLPH, V. & 8 others (1979). *Cis*-dichlorodiammineplatinum (II) based chemotherapy as initial treatment of advanced head and neck cancer. *Cancer Treat. Rep.*, **63**, 1533.

WOLF, G.T., MAKUCH, R.W. & BAKER, S.R. (1984). Predictive factors for tumour response to preoperative chemotherapy in patients with head and neck squamous carcinoma. *Cancer*, **54**, 2869.

UICC (1987). *Classification of Malignant Tumours*, 4th edn. Springer-Verlag: Berlin.